# Depression increased risk of coronary heart disease: A meta-analysis of prospective cohort studies

**DOI:** 10.3389/fcvm.2022.913888

**Published:** 2022-08-30

**Authors:** Hongfu Cao, Hui Zhao, Li Shen

**Affiliations:** ^1^Gulou Hospital of Traditional Chinese Medicine of Beijing, Beijing, China; ^2^Institute of Basic Theory for Chinese Medicine, China Academy of Chinese Medical Sciences, Beijing, China

**Keywords:** depression, coronary heart disease, relative risk, meta-analysis, prospective cohort studies

## Abstract

**Background:**

Depression, as an independent risk factor, can lead to a substantially increased risk of coronary heart disease (CHD). The overall body of evidence involving depression and CHD is not consistent. Therefore, we performed an update meta-analysis to evaluate the association between depression and the risk of patients with CHD.

**Methods:**

Studies were identified through a comprehensive literature search of the PubMed, Embase, and the Cochrane Library database from its inception to 28 September 2021 for titles/abstracts with restricted to English language articles. The literature was screened according to the inclusion and exclusion criteria. Along with data extraction, we evaluated the quality of eligible studies using the Newcastle-Ottawa Scale (NOS). The primary outcome was fatal or non-fatal CHD. We calculated relative risk (RR) with 95% confidence intervals (CIs) using a random-effects models. The protocol was registered in the PROSPERO registration (registration number CRD42021271259).

**Results:**

From 9,151 records, we included 26 prospective cohort studies published from 1998 to 2018, consisting of 402,597 patients. Either in depression-exposured group or non-depression-exposured group, the mean age of all participants ranged from 18 to 99 years. Moreover, the NOS scores of these studies are eventually indicated that the quality of these eligible studies was reliable. In general, the pooled results showed that patients with depression had a higher risk of CHD compared to patients without depression (RR = 1.21, 95% CI: 1.14–1.29). Additionally, the funnel plot appeared to be asymmetry, indicating there existing publication bias for the pooled results between depression and CHD. A sensitivity analysis was used to assess the stability of the relationship between depression and CHD that indicating the results robust (RR = 1.15, 95% CI: 1.09–1.21).

**Conclusion:**

Depression may increase risk of CHD. Future studies on the share pathogenic mechanisms of both depression and CHD may develop novel therapies.

## Introduction

Coronary heart disease (CHD) is the most common heart disease worldwide, accounting for an estimated 200 million people (1). In 2019, the World Health Organization estimated that approximately 17.9 million of worldwide deaths were due to CHD (2). It is a chronic and complex disease that refers to a general term for disease narrowing of the coronary artery wall owing to fatty material accumulation (3). Its primary clinical symptoms represented chest pain, chest tightness, syncope, and so on, posing a severe effect to human health and the quality of life (4, 5). The growing burden of CHD has been a main public health problem in China (6). Despite the pathogeny of CHD is not entirely clear, risk factors such as age, gender, obesity, diabetes, high cholesterol, high blood pressure, and unhealthy living habits like smoking, could explain about 50–60% of the etiology of CHD (7–9).

Depressive is a leading and growing cause of disability, a main contributor to the total global burden of disease, with more than an estimated 280 million people affected worldwide (10). Depression is considered a chronic disease affecting 26% women and 18% men (11). The psychological symptoms that patients with depression experience are the primary contributing factors to its heavy disease burden (12). The burden posed by depression could be alleviated by increasing access to timely treatment. Depression results from a complex interaction of various factors. At present, the relationship between depression and CHD has attracted increasing academic attention. According to position papers of the American Heart Association and the European Society of Cardiology, depression may be a modifiable prognostic factor for CHD (13, 14). Apart from those, it has been reported that depression, as an independent risk factor, can lead to a substantially increased risk of CHD, as it not only diminished the quality of life in patients with CHD, but also increased the occurrence rate of main adverse cardiac events (15, 16).

Although a number of studies showed a specific link between depression and CHD (17–19), the overall body of evidence is not consistent (20, 21). Meta-analysis overcomes the shortcomings of traditional literature review, and has the characteristics of quantitative synthesis; it can provide a systematic, repeatable and objective synthesis method for the same problem (22). In the previous meta-analysis (23) an increased risk of myocardial infarction (MI) and coronary death was found with the presence of depression in people. The study however assessed 19 studies including two diseases of MI and CHD from 1996 to 2014. Therefore, in this present study, we aim to perform an update the meta-analysis and further the nature of the relationship between depression and CHD morbidity.

## Materials and methods

### Literature and search strategy

We adhered to the Preferred Reporting Items for Systematic Reviews and Meta-Analyses (PRISMA) guidelines to perform this updated systematic review and meta-analysis to determine the relationship between depression and CHD.(24) The protocol was registered in the PROSPERO registration (registration number CRD42021271259). Studies were identified through a comprehensive literature search of the PubMed, Embase, and the Cochrane Library database from its inception to 28 September 2021 for titles/abstracts with restricted to English language articles, by two authors independently. The following search term of the Medical Subject Headings (MeSH)/Emtree combined with free text words as well as all known spellings were used to search: depression/depression disorder and Coronary Disease, which could be adjusted in different databases. Detailed full-search strategies were provided in Supplementary Table 1. Moreover, no restriction in region, publication years and types were in this literature search. A further complementary screening of the references of eligible published studies and meta-analysis was tracked manually as a supplementation.

### Selection criteria

Published studies were included if they simultaneously could meet the following criteria. These studies: (1) recruited subjects who were depression without CHD at baseline visit; (2) they were exposed to depression (yes or not); moreover, depression was defined by diagnostic interview, self-completed scaled questionnaire, anti-depressant medication, physician diagnosis, or self-reported diagnosis; (3) studies provided relative risk (RR), hazard ratio (HR), or odds ratio (OR) with 95% confidence interval (CI); and (4) they were adopted prospective cohort study design with predictors’ assessment at baseline. When one study met one of the following criteria, it was excluded: (1) they were unable to obtain original text, such as conference abstract, review article etc.; (2) they included other types of diseases not as a single outcome; (3) the required data cannot be calculated; and (4) only non-desired outcomes were reported. For studies with the same data in at least two studies, we give priority to including the most recent and detailed studies. If there were divergences between two authors, these studies were discussed in further detail or arbitrated by third author until agreement was reached.

### Data extraction and quality assessment

The following information was extracted independently by two authors detailing with a predesigned Excel spreadsheet carefully from each study, including the first author’s name, publication year, study design, population size, country, age, female (%), race, diabetes, hypertension, history of smoking, hyperlipidemia, BMI categories, depression measurement, outcomes, the adjusted and unadjusted effect estimates (HR, RR, or OR) with CI, adjustment variables included in the final model, and length of follow-up. For studies provided both adjusted and unadjusted effect estimates (HR, RR, or OR), the adjusted one was used to further analysis.

Along with data extraction, we evaluated the quality of eligible studies using the Newcastle-Ottawa Scale (NOS) (25), which contains eight items classified into three domains: selection, comparability, and outcome. The NOS scores ranged from 1 to 9 “★,” with 9 “★” representing the best quality. Data extraction and quality assessment were conducted by two authors independently, and if there exists any disagreement, a third author resolved and provided an arbitration.

### Statistical analysis

The primary outcome was the confounder adjusted HR, OR, and RR with the corresponding 95% CI. The original HRs obtained in the text were considered as equivalent estimations for RRs.(26) For a study only reported OR, particular formulae were used to convert OR to RR.(27) We calculated the pooled RR with the corresponding 95% CI for the association between depression and the risk of CHD. The heterogeneity between studies was quantified using Cochrane *Q*-test and the *I*^2^ statistic. The statistically significant refers to *Q* ≤ 0.1 or *I*^2^ > 50% (28). When there existed significant heterogeneity, a random-effects model was selected, if not, we chose the fixed-effect model to perform the pooled analyses. In addition, subgroup analyses were conducted to explore potential sources of heterogeneity based on age, sex, publication year, outcome indicator, and duration of follow-up. Subsequently, we performed sensitivity analysis by excluding one study in turn to assess the robustness of the meta-analysis results. Furthermore, funnel plots, Begg’s test and Egger’s test were used to evaluate potential publication bias, which was defined as *P* < 0.05. All mentioned above data analyses were performed with Stata 14.0 software (STATA Corp., College Station, TX, United States).

## Results

### Literature search and selection

**FIGURE 1:**
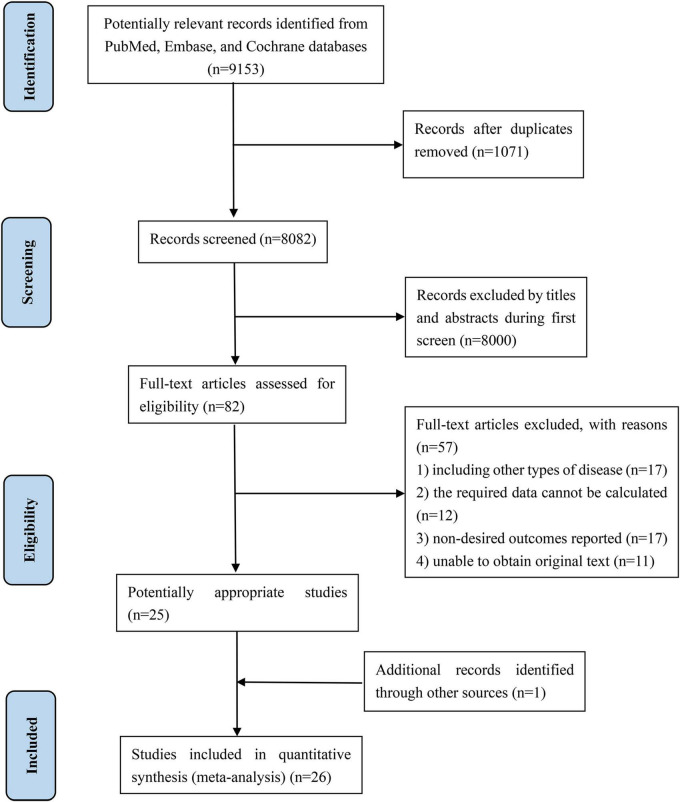
Flowchart for searching and selection details.

The database search and literature selection are shown in Figure 1. Overall, 9,153 papers were retrieved *via* the initial literature search, among which 1,071 were removed due to duplication. Subsequently, after title and abstract screening, 8,000 papers were further excluded, remaining 82 potentially eligible papers for full-text review. After reviewing the full-text articles, a total of 25 papers were included into this present meta-analysis since 17 of them include other types of disease, 12 cannot calculated the required data, 17 did not report desired outcomes, and 11 were unable to obtain original text. Moreover, we also manually tracked the references of retrieved papers and meta-analyses, and an additional paper has been added. Finally, a total of 26 articles (20, 21, 29–52) were included in the systematic review and meta-analysis.

### Characteristics and quality assessment of the included eligible studies

Among included 26 prospective cohort studies, the publication year ranged from 1998 to 2018 and the sample capacity included 402,597 subjects. With respect to the regions that conducted these studies, the majority of them was conducted in one country or region, two of them in several countries, and another study that did not indicate where it was done. Either in depression-exposured group or non-depression-exposured group, the mean age of all participants ranged from 18 to 99 years. The measurement of depression mainly involved Center for Epidemiological Studies-Depression (CES-D), International Classification of Diseases, Seventh, Eighth, Ninth, Tenth Revision (ICD-7, -8, -9, -10), Geriatric Depression Scale (GDS), Beck Depression Inventory (BDI), Mental Health Index-5 (MHI-5), Zung Self-rating Depression Scale (ZSDS), Minnesota Multiphasic Personality Inventory (MMPI-2 D), Structured Clinical Interview for Diagnostic and Statistical Manual of Mental Disorders, Fourth Edition, Non-patient edition (SCID-I/NP), and diagnostic and statistical manual of mental disorders, third edition (DSMMD). Nineteen studies reported outcome indicators using HR, whereas five reported using RR, and two reported using OR. Notably, the follow-up time for the prospective cohort study were reported, ranging from 4 to 37 years. All included studies evaluated outcomes using multiple adjustment variables, however, one study only have adjusted for age.

As described previously, we used the NOS to evaluate the 26 included studies. Detailed scores are presented in Supplementary Table 2, in which “★” representing 1 point, “×” representing 0 point, and “—” representing uncertain points. Moreover, the quality scores of these studies are eventually presented in Table 1, the scores were 6 to 9 “★,” indicating that the quality of these studies was reliable.

**TABLE 1:** Characteristics of studies included in the meta-analysis.

References, region	Sample	Female/ male (*N*)	Age [years, *M* (SD)]	Depression measurement	Outcome	Duration of follow-up	Risk type	Adjustment variables	NOS
Hawkins et al. (49), Indianapolis	2,537	296/90	66.1	CES-D	CAD	15 years	HR	Adjusted for age, sex, race, diabetes, hypertension, smoking, hyperlipidemia, and excess body weight.	8
		1,516/635	67.6						
Brunner et al. (48), London	31,395			CES-D	CHD	24 years	HR	Adjusted for age, sex, and ethnicity.	7
Rahman et al. (46), Swedish	36,654	336/243	63	ICD–7, –8, –9, –10	CHD	4 years	HR	Adjusted for birth year, sex, smoking status, educational level, hypertension, diabetes, alcohol intake, and BMI.	7
		15,651/14,141	63						
Huang et al. (44), Taiwan	39,685	4,966/2,971	20–99	ICD-9	CHD	9 years	HR	Adjusted for age, sex, diabetes mellitus, hypertension, hyperlipidemia, alcohol-related illness, obesity, COPD, influenza vaccinations, and cardiology visits.	8
		19,864/11,884							
Sun et al. (47), Hong Kong	62,839	4,545/1,514	≥65	GDS	CHD	8 years	HR	Adjusted for age, education, monthly expenditure, smoking, alcohol drinking, physical activity, BMI, and sex.	9
		36,779/19,943							
Péquignot et al. (45), France	7,308	1,306/357	74.3 (5.5)	CES-D	CHD	6 years	HR	Adjusted for age, study center, and sex, smoking status, alcohol consumption, high BP, impaired fasting glycemia or diabetes, hypercholesterolemia, living alone, education level, and MMSE score.	7
		3,334/2,317	73.6 (5.3)						
Majed et al. (43), France and Northern Ireland	9,601	–/1,979	54.76 (2.81)	CES-D	CHD	10 years	HR	Adjusted for age, study centers, and socioeconomic factors, including marital status, education level, employment status, physical activity, smoking status, daily alcohol intake, SBP, use of anti-hypertensive drugs, BMI, total and high-density lipoprotein cholesterol, treatment for diabetes, and use of antidepressant treatment.	7
		–/6,767	50–59						
Brown et al. (42)	2,728	326/97	66.1 (6.5)	CES-D	CHD	13–16 years	RR	Adjusted for age, sex, race, diabetes, hypertension, history of smoking, cholesterol, and ideal body weight.	7
		1,623/682	67.7 (7.0)						
Janszky et al. (40), Swedish	49,321	–/646	18–20	ICD-8	CHD	37 years	HR	Adjusted for smoking, body length, diabetes, SBP, alcohol consumption, physical activity, father’s occupation, family history of CHD, and geographic area.	6
		–/48,675							
Nabi et al. (41), Finnish	23,282	3,031/1,673	20–54	BDI	CHD	7 years	HR	Adjusted for sex, age and education, alcohol consumption, sedentary lifestyle and smoking, obesity, hypertension or diabetes, and incident CHD or incident CBVD.	9
		10,744/7,834							
Davidson et al. (38), Nova Scotia	1,794	899/895	46.3 (18.3)	CES-D	CHD	>10 years	HR	Adjusted for sex, age, and Framingham risk score.	7
Whang et al. (39), Massachusetts	63,469	0–52: 4,994/–	0–52: 56.7 (7.4)	MHI-5	Fatal CHD	8 years	HR	Adjusted for age, beginning year of follow-up, smoking status, BMI, alcohol intake, menopausal status and postmenopausal hormone use, usual aspirin use, multivitamin use, vitamin E supplement use, hypercholesterolemia, family history of MI, history of stroke, n-3-fatty acid intake, alpha linolenic acid intake, and moderate/vigorous physical activity, with the addition of non-fatal CHD during follow-up, hypertension, and diabetes.	7
		53–75: 7,030/– 76–85: 2814/– 86–100: 18,631/–	53–75: 57.6 (7.2) 76–85: 58.3 (7.2) 86–100: 59.6 (7.1)						
Ahto et al. (37), Finland	1,360	62/38	F: 72.9 (5.8) M: 71.5 (6.2)	ZSDS	CHD	12 years	HR	Adjusted for age, occurrence of symptoms of depression, marital status, social status, and number of medicines as possible predictors.	7
		316/244	F: 71.3 (5.8) M: 71.1 (6.3)						
Kamphuis et al. (36), Finland, Italy, Netherlands	799	–/799	70–90	ZSDS, CES-D	CHD	10 years	HR	Adjusted for country, education, BMI, smoking, alcohol intake, SBP, total and high-density lipoprotein cholesterol levels, and physical activity.	8
Wulsin et al. (35), Framingham	3,634	348/164	50 (13)	CES-D	CHD	5.9 years	HR	Adjusted for sex stratified, adjusted for age, smoking, hypertension, diabetes, BMI, total cholesterol, and alcohol consumption.	7
		1,655/1,467	50 (14)						
Gump et al. (33), United States cities	12,866	–/12,866	35–57	CES-D	CHD	18.43 years	HR	Adjusted for age, intervention group, race, educational attainment, smoking at baseline and visit 6, trial averaged SBP, alcohol consumption, and fasting cholesterol, as well as the occurrence of non-fatal cardiovascular events during the trial.	9
Marzari et al. (34), Italian	2,830	712/440	≥65	GDS-30	CHD	4 years	HR	Adjusted for age, marital status, years of schooling, smoking status, drinking status, depressive symptomatology, ADL disability level, fibrinogen, platelets, total cholesterol, triglycerides, glycemia, BMI, arrhythmia, hypertension, diabetes, congestive heart failure, claudication, stroke, and dementia.	6
		668/1,010	F: 73.0(5.7) M: 73.0(5.5)						
Ferketich et al. (32), United States	7,893	874/280	F: 53.7 (13.9) M: 55.9 (14.4)	CES-D	CHD	8.3 years	RR	Adjusted for poverty index, smoking, race, hypertension, diabetes, BMI, and non-fatal CHD events.	8
		4,132/2,617							
Sesso et al. (31), Boston	2,280	–/2,280	21–80	MMPI-2 D	CHD	7 years	RR	Adjusted for age, smoking status, systolic and diastolic BP, BMI, family history of CHD, and alcohol intake.	8
Jiang et al. (52)	998	998/–	<65: 62.0 (7.04) ≥65: 79.58 (4.97)	GDS	CHD	9 years	OR	Adjusted for age.	6
			<65: 60.87 (7.75) ≥65: 81.21 (4.98)						
Mendes De Leon et al. (30), New Haven, CT, United States	2,391	1,446/945	65–99	CES-D	CHD	9 years	RR	Adjusted for age, CHD risk factor, and physical functioning.	8
Péquignot et al. (21), French	7,313	2,170/669	≥65	CES-D	CHD	10 years	HR	Adjusted for age, sex, city, education level, living alone, current smoking, >3 glasses of alcohol a day, diabetes mellitus, hypertension, hypercholesterolemia, and MMSE.	9
		2,474/2,000	73.4 (5.3)						
O’Neil et al. (51), South-Eastern Australia	860	148/–	46.4 (13.5)	SCID-I/NP	CHD	18 years	OR	Adjusted for family history of CHD, age, years of smoking, SBP and total and HDL cholesterol level, anxiety due to the overlap between depression and anxiety disorder, atypical including albumin, high-sensitivity C-reactive protein, education levels, diastolic BP, pulse rate, BMI, LDL cholesterol, 10 year changes in mobility, alcohol use, triglycerides, and medication use.	6
		713/–	48.3 (16.1)						
Sims et al. (50), United States	24,261	1,848/682	62.5 (9.7)	CES-D	CHD	4.2 (1.5) years	HR	Adjusted for age, sex, marital status, region, race, education, residence in census tract with poverty level of 30% or more, physical activity, smoking, alcohol consumption, diabetes, BMI, log transformed hsCRP, SBP, TC, HDL, log transformed urinary albumin to creatinine ratio, triglycerides, use of anti-hypertensive medication, and use of statins.	8
		12,330/9,401	64.3 (9.3)						
O’Brien et al. (20), Jackson, MS, United States	3,309	536/202	52.2 (42.9–63.0)*	CES-D	CHD	>10 years	HR	Adjusted for CHD risk score, SES, behavioral risk factors, antidepressant use, and coping strategies.	8
		1,627/944	54.3 (44.9–63.7)						
Ford et al. (29), United States	1,190	–/132	27 (3.0)	DSMMD	CHD	26 years	RR	Adjusted for graduation age, baseline serum cholesterol level, premature parental myocardial infarction, physical activity, time-dependent smoking, incident hypertension, and incident diabetes.	6
		–/1,058	26 (2.4)						

F, female; M, male; GDS, Geriatric Depression Scale; BDI, Beck Depression Inventory; MHI, Mental Health Index; ZSDS, Zung Self-rating Depression Scale; SCID-I/NP, Structured Clinical Interview for Diagnostic and Statistical Manual of Mental Disorders, Fourth Edition, Non-patient edition; CES-D, Center for Epidemiological Studies-Depression; DSMMD, diagnostic and statistical manual of mental disorders, third edition; SBP, systolic blood pressure; MMSE, Mini Mental State Examination; TC, Total Cholesterol. *Median (IQR).

### Meta-analysis of the association between depression and coronary heart disease

**FIGURE 2:**
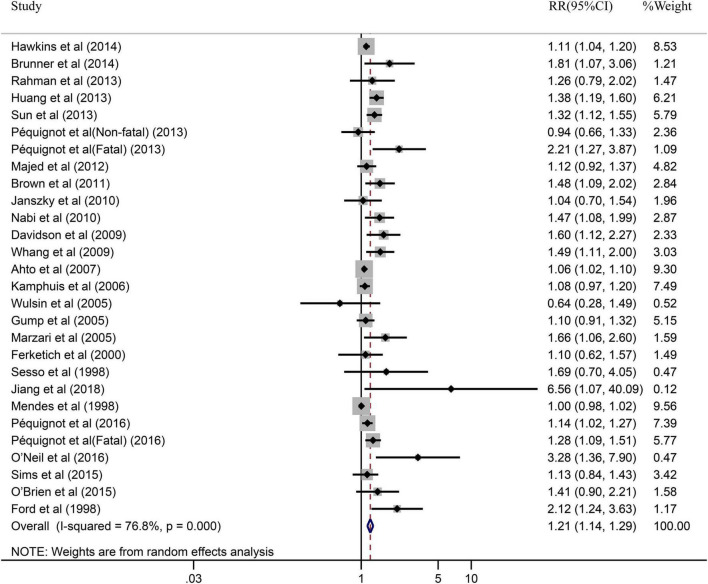
Forest plot presents the association between depression and the risk of CHD in prospective cohort study.

The impact of depression on CHD was evaluated in 28 cohort studies, due to fatal and non-fatal being divided into 2 separate studies. Figure 2 illustrated the results of pooled RR with a random-effects model. In general, the pooled results showed that patients with depression had a higher risk of CHD compared to patients without depression (RR = 1.21, 95% CI: 1.14–1.29). Simultaneously, the heterogeneity was significant (*I^2^* = 76.8%, *P* = 0.000). Additionally, we also performed a sensitivity analysis to assess the stability of our results regarding the relationship between depression and CHD. Omitting one study at a time by sequentially, we found no significant changes, indicating the results robust (RR = 1.15, 95% CI: 1.09–1.21) (shown in Supplementary Figure 1).

### Subgroup analysis

**FIGURE 3:**
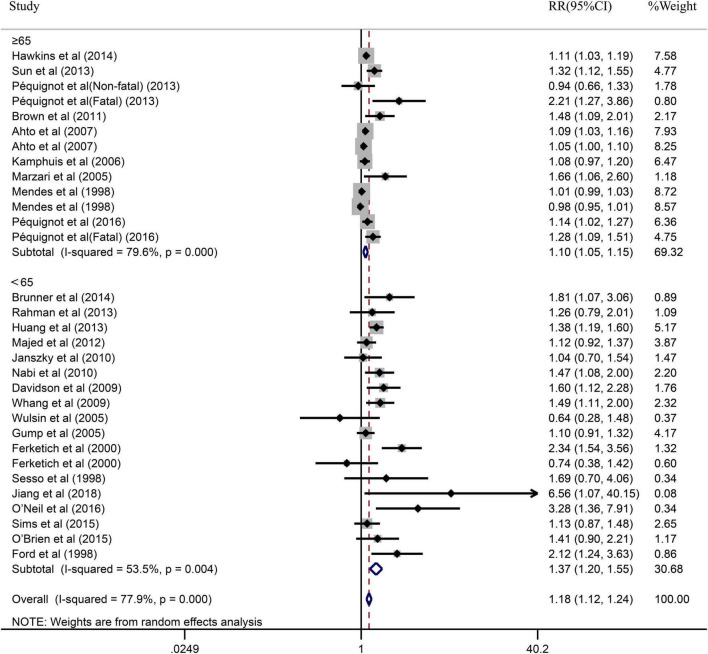
Subgroup analysis with age (≥65 or <65) presents the association between depression and the risk of coronary heart disease in a prospective cohort study.

**FIGURE 4:**
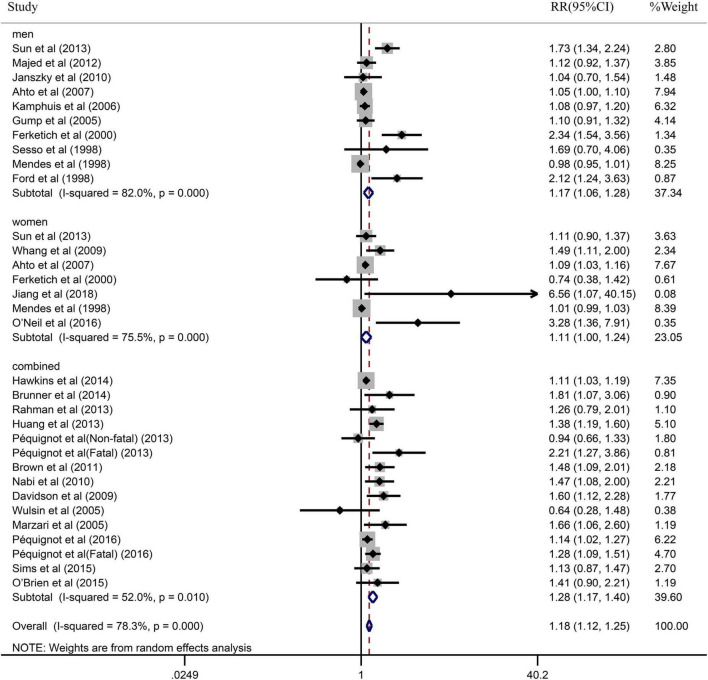
Subgroup analysis with sex (men, women, or combined) presents the association between depression and the risk of coronary heart disease in a prospective cohort study.

**FIGURE 5:**
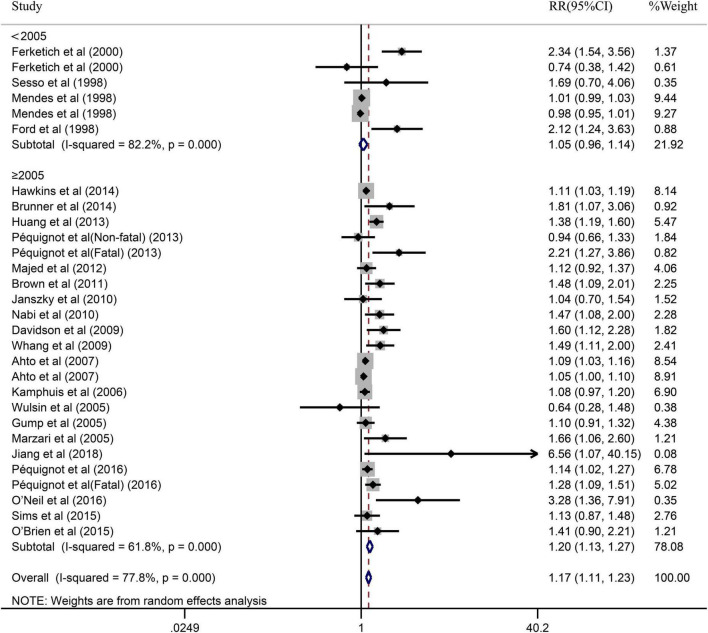
Subgroup analysis with publication year (<2005 or ≥2005) presents the association between depression and the risk of coronary heart disease in a prospective cohort study.

**FIGURE 6:**
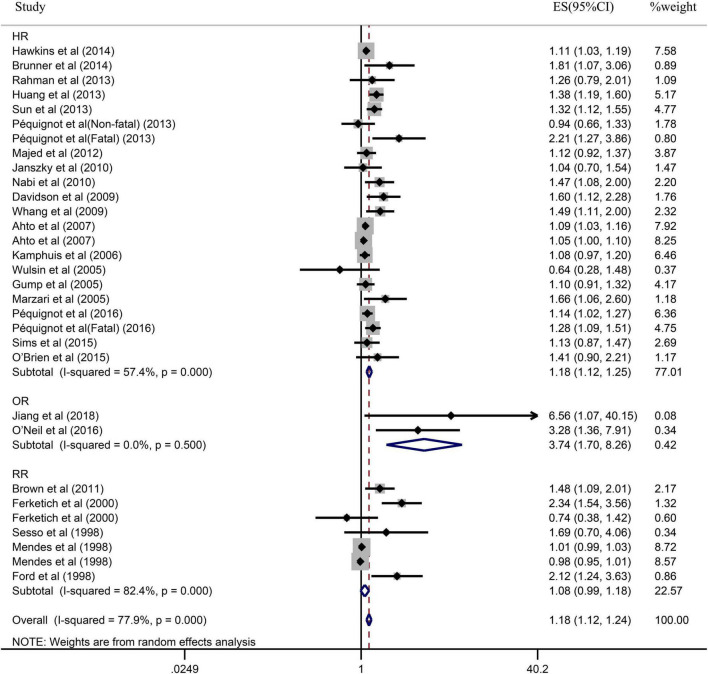
Subgroup analysis with evaluation index (HR, OR, or RR) presents the association between depression and the risk of coronary heart disease in a prospective cohort study.

**FIGURE 7:**
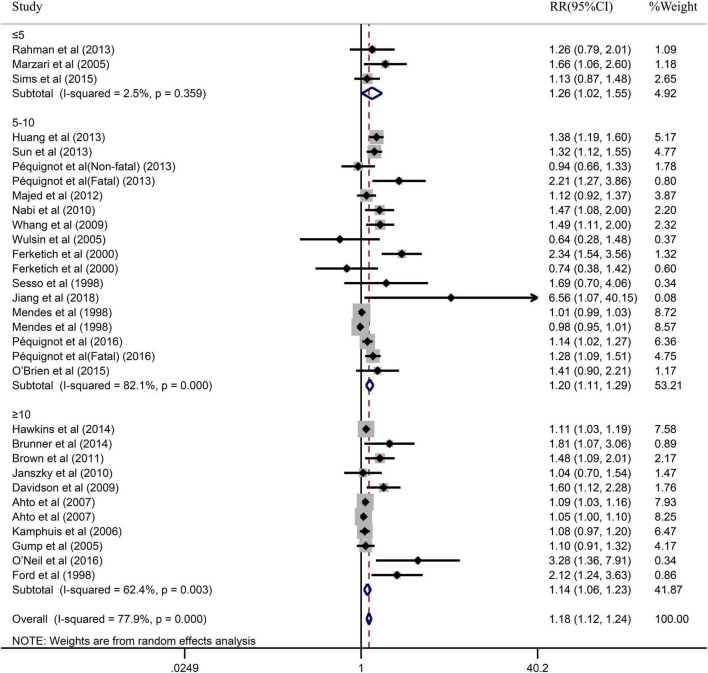
Subgroup analysis with the duration of follow-up (≤5, 5–10 or ≥10) presents the association between depression and the risk of coronary heart disease in a prospective cohort study.

Due to the significant heterogeneity of the pooled results, subgroup analysis was further conducted to explore the source of the heterogeneity. Results of the subgroup analysis by age (≥65 or <65) indicated that the pooled RR were 1.10 (95% CI: 1.05–1.15) in ≥65 studies and 1.37 (95% CI: 1.20–1.55) in <65 studies. However, the intragroup heterogeneity did not decrease significantly (Figure 3). In addition, based on either sex (men, women, or combined) or publication (≥2005 or <2005), no statistically significant association was observed (present respectively in Figures 4, 5). Furthermore, a stratified subgroup analysis based on evaluation index was found that the pooled results were 1.18 (95% CI: 1.12–1.25) in HR group, 3.74 (95% CI: 1.70–8.26) in OR group, and 1.08 (95% CI: 0.99–1.18) in RR group. Besides, the intragroup heterogeneity decreased to a low level of 0% in OR group (*P* = 0.05) (Figure 6). Similarly, the statistically significant association was observed in ≤5 follow-up group (Figure 7).

### Publication bias

**FIGURE 8:**
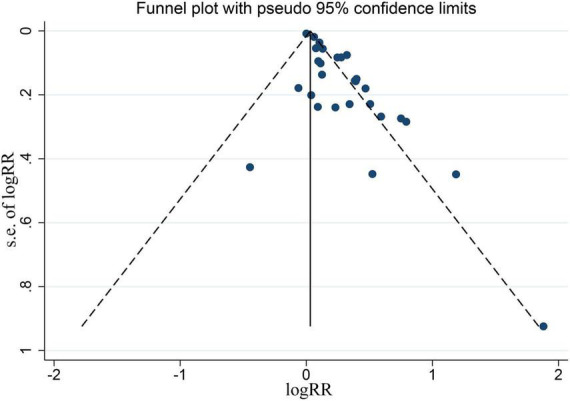
Funnel plot for all the eligible studies that provided ORs, RRs, or HRs for depression and the risk of CHD.

The funnel plot appeared to be asymmetry, indicating there existing publication bias for the pooled results between depression and CHD, as indicated Figure 8. In the meanwhile, the Begg’s rank correlation and Egger linear regression test also documented significant (*P* < 0.05), as shown in Supplementary Figures 2, 3.

## Discussion

This present study updated the meta-analysis of the risk association between depression and CHD based on a broad range of studies involving 402,597 participants. Our results are limited to prospective cohort studies suggest that depression may be modifiable risk factor for CHD. Moreover, the increased risk related with depression persisted and remained statistically significant in sensitivity analysis when eliminating the studies one by one as well as in all subgroup analysis stratified by participant age, sex, and follow-up. In the meanwhile, we also found that the publication bias of this study was significant. Due to the worldwide prevalence of depression, our findings of the meta-analysis have significant meanings for global public health.

Increased risk between depression and CHD was found in the previous several meta-analyses, which is consistent with our study findings (17, 19, 23). However, these studies were published relatively early, which could not include more recent literatures, which is likely to decrease the credibility of the evidence. In addition, of which, one study (23) also included MI not just simple CHD. Therefore, on the basis of previous meta-analyses, our study also included literatures of recent years, indicating that depression was an important risk factor for CHD, which further makes the results more credible.

In our meta-analysis, there exist different degrees of heterogeneity for the relationship between depression and CHD. According to the subgroup analyses, some significant and valuable results were identified. A major finding was that depression could increase the risk of CHD in the group of evaluation index using RR, whereas was significant in the group of evaluation index using HR and OR. The RR did not take time into account when assessing depression increased risk of CHD, and as well known, depression could be treatable and is strongly correlated with the duration of follow-up. In the meantime, we also found that there was no statistically significant in the group of studies published 2005 year and later, but did not have statistically significant relationship for studies published 2005 year and before. One possible explanation to this finding was that might be due to the small number of included studies published before 2005.

The specific mechanisms connecting depression to increased CHD risk are complex and multifactorial, which were still incompletely clear until now (53). One possible explanation is chronic dysregulation of autonomic function, refers to an imbalance of the sympathetic and parasympathetic systems, which is considered to be a crucial mechanism (53). Previous animal study has shown that depression may result with cardiovascular and autonomic imbalance presenting elevating heart rate, reducing heart rate variability (54), and elevating cardiac sympathetic tone (55, 56). Furthermore, the majority studies of patients with CHD have found that compared with those without depression, patients with depression have lower HRV and higher heart rate, and decreased baroreceptor sensitivity as well as increased QT interval variability and heart rate turbulence (53, 57). Another hypothesized explanation for the increased risk of CHD related with depression is chronic inflammation that is a well-known risk factor for CHD occurrence and development (58). In addition to the mechanisms mentioned above, previous studies also have proposed the following underlying mechanisms, such as changed brain and neuronal function affecting neuroendocrine pathways, immune responses, life behavior, platelet activation and thrombosis, as well as cardiac metabolic risk factors (14, 57). The available evidence have indicated that depression consistently associated with increased risk of CHD (17, 23, 57) and vice versa (59, 60). Not only that, but related studies have reported that two conditions are highly comorbid (61, 62). Psychopathy regulation could obviously improve the quality of life of patients with CHD and decrease the incidence rate of acute cardiovascular events (63).

### Limitations

Certainly, there are also a few limitations should be acknowledged in this current meta-analysis, despite our results are promising. Firstly, in this meta-analysis, we only included studies mainly in English, which may cause publication bias. Therefore, we adopted the search strategy of the Mesh combined free keyword to make the retrieval as comprehensive as possible to eliminate publication bias as much as possible. Secondly, another possible limitation of the current meta-analysis is the use of various methods to assess depression. We try to ensure included eligible studies with the same evaluation method to reduce heterogeneity. Thirdly, considerable heterogeneity existed in our study, to which we applied a random-effects model when a significant heterogeneity is existing; therefore, the increased risk of depression for CHD may be underestimated. Finally, confounding factors of outcome evaluation indexes were inconsistent, which may produce biases. Despite risk factors have been adjusted using multivariate, the possibility of other inadequate adjustment for unmeasured or imprecisely measured confounding factors that increase the risk of CHD cannot be excluded.

## Conclusion

In summary, in a meta-analysis of 402,597 participants in 26 prospective cohort studies, depression may increase risk of CHD. Future studies on the share pathogenic mechanisms of both depression and CHD may develop novel therapies. The clinical significance lies in raising vigilance against patients with depression and CHD, and early detection and timely medical treatment for CHD patients through a multiple approach.

## Data availability statement

The original contributions presented in this study are included in the article/Supplementary material, further inquiries can be directed to the corresponding author.

## Author contributions

HC and LS: conceptualization, methodology, and software. HC and HZ: data curation and writing—original draft preparation. LS: visualization, investigation, supervision, validation, and writing—reviewing and editing. All authors contributed to the article and approved the submitted version.

## Supplementary material

Supplementary Figure 1 Sensitivity analysis for all the eligible studies that provided ORs, RRs, or HRs for depression and the risk of CHD.

Supplementary Figure 2 Begg’s funnel plot for all the eligible studies that provided ORs, RRs, or HRs for depression and the risk of CHD.

Supplementary Figure 3 Egger’s publication bias plot for all the eligible studies that provided ORs, RRs, or HRs for depression and the risk of CHD.

Supplementary Table 1 Detailed full-search strategies in different databases.

Supplementary Table 2 Detailed scores of NOS for all eligible studies.
