# Supplementary figures and images for: Depression increased risk of coronary heart disease: A meta-analysis of prospective cohort studies
Source: Front Cardiovasc Med. 2022 Aug 30;9:913888. doi: 10.3389/fcvm.2022.913888 (PMC9468274; doi:10.3389/fcvm.2022.913888)

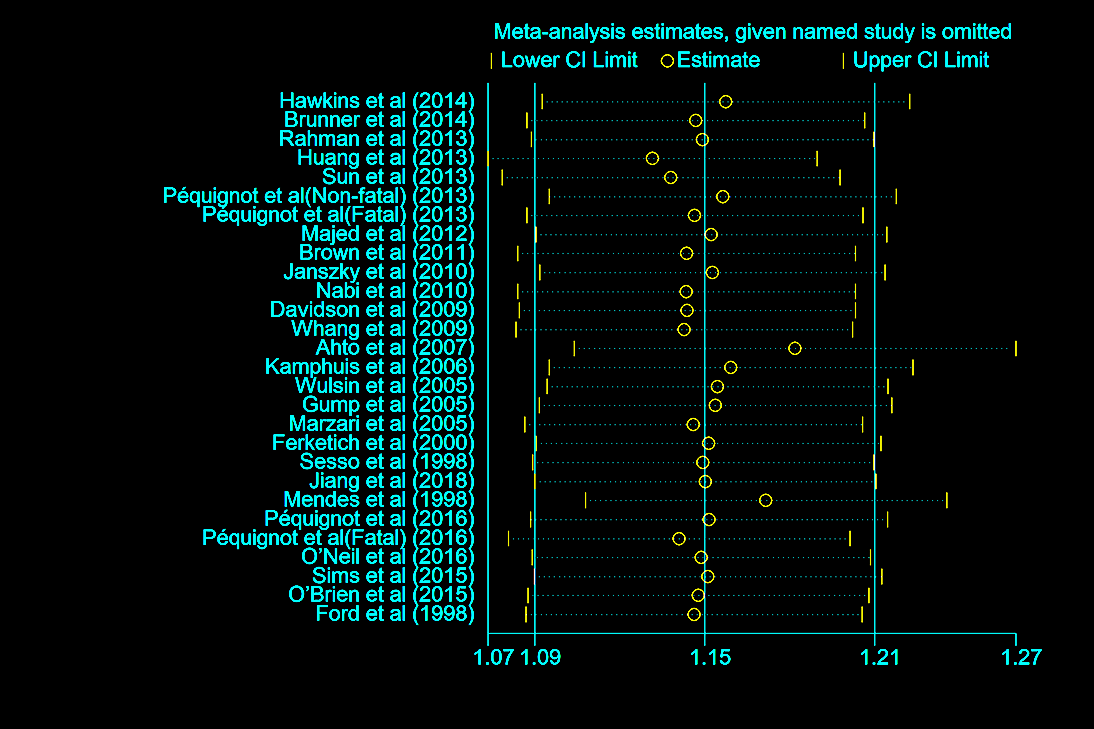

Supplement: Supplementary Figure 1 — Sensitivity analysis for all the eligible studies that provided ORs, RRs, or HRs for depression and the risk of CHD. [file Image_1.TIF]

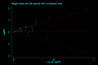

Supplement: Supplementary Figure 2 — Begg’s funnel plot for all the eligible studies that provided ORs, RRs, or HRs for depression and the risk of CHD. [file Image_2.TIF]

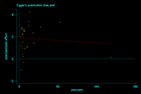

Supplement: Supplementary Figure 3 — Egger’s publication bias plot for all the eligible studies that provided ORs, RRs, or HRs for depression and the risk of CHD. [file Image_3.TIF]
