# Supplementary material for: Depression increased risk of coronary heart disease: A meta-analysis of prospective cohort studies
Source: Front Cardiovasc Med. 2022 Aug 30;9:913888. doi: 10.3389/fcvm.2022.913888 (PMC9468274; doi:10.3389/fcvm.2022.913888)
Supplement: Supplementary Table 1 — Detailed full-search strategies in different databases. [file Table_1.DOCX]

| Supplementary 1  Relevant databases retrieval record | | | |
| --- | --- | --- | --- |
| Source: PubMed (Searched on: 28 September 2021) | | | |
| Search number | | Search Details | Results |
| #54 | | #42 OR "depressions endogF AND #53 | 2,429 |
| #53 | | #43 OR #44 OR #45 OR #46 OR #47 OR #48 OR #49 OR #50 OR #51 OR #52 | 250,423 |
| #52 | | "heart diseases coronary"[Title/Abstract] | 17 |
| #51 | | "heart disease coronary"[Title/Abstract] | 248 |
| #50 | | "diseases coronary heart"[Title/Abstract] | 97 |
| #49 | | "disease coronary heart"[Title/Abstract] | 284 |
| #48 | | "Coronary Heart Diseases"[Title/Abstract] | 971 |
| #47 | | "Coronary Heart Disease"[Title/Abstract] | 52,128 |
| #46 | | "diseases coronary"[Title/Abstract] | 214 |
| #45 | | "disease coronary"[Title/Abstract] | 1,375 |
| #44 | | "Coronary Diseases"[Title/Abstract] | 659 |
| #43 | | "Coronary Disease"[MeSH Terms] | 225,167 |
| #42 | | #13 OR #41 | 261,188 |
| #41 | | #14 OR #15 OR #16 OR #17 OR #18 OR #19 OR #20 OR #21 OR #22 OR #23 OR #24 OR #25 OR #26 OR #27 OR #28 OR #29 OR #30 OR #31 OR #32 OR #33 OR #34 OR #35 OR #36 OR #37 OR #38 OR #39 OR #40 | 122,404 |
| #40 | | "Unipolar Depressions"[Title/Abstract] | 38 |
| #39 | | "depressions unipolar"[Title/Abstract] | 3 |
| #38 | | "depression unipolar"[Title/Abstract] | 58 |
| #37 | | "Unipolar Depression"[Title/Abstract] | 2,881 |
| #36 | | "Melancholias"[Title/Abstract] | 12 |
| #35 | | "Melancholia"[Title/Abstract] | 1,484 |
| #34 | | "Neurotic Depressions"[Title/Abstract] | 46 |
| #33 | | "Neurotic Depression"[Title/Abstract] | 347 |
| #32 | | "Depressions, Neurotic"[Title/Abstract] | 0 |
| #31 | | "Depressions, Neurotic"[Title/Abstract] | 0 |
| #30 | | "depression neurotic"[Title/Abstract] | 27 |
| #29 | | "syndromes depressive"[Title/Abstract] | 11 |
| #28 | | "syndrome depressive"[Title/Abstract] | 25 |
| #27 | | "Depressive Syndromes"[Title/Abstract] | 860 |
| #26 | | "Depressive Syndrome"[Title/Abstract] | 1,094 |
| #25 | | "Endogenous Depressions"[Title/Abstract] | 224 |
| #24 | | "Endogenous Depression"[Title/Abstract] | 1,544 |
| #23 | | "depressions endogenous"[Title/Abstract] | 2 |
| #22 | | "depression endogenous"[Title/Abstract] | 21 |
| #21 | | "neuroses depressive"[Title/Abstract] | 2 |
| #20 | | "Depressive Neurosis"[Title/Abstract] | 147 |
| #19 | | "Depressive Neuroses"[Title/Abstract] | 37 |
| #18 | | "neurosis depressive"[Title/Abstract] | 5 |
| #17 | | "disorders depressive"[Title/Abstract] | 242 |
| #16 | | "disorder depressive"[Title/Abstract] | 267 |
| #15 | | "Depressive Disorders"[Title/Abstract] | 10,881 |
| #14 | | "Depressive Disorder"[MeSH Terms] | 114,503 |
| #13 | | #1 OR #2 OR #3 OR #4 OR #5 OR #6 OR #7 OR #8 OR #9 OR #10 OR #11 OR #12 | 166,788 |
| #12 | | "Emotional Depressions"[Title/Abstract] | 0 |
| #11 | | "Emotional Depressions"[Title/Abstract] | 0 |
| #10 | | "Depressions, Emotional"[Title/Abstract] | 0 |
| #9 | | "Depressions, Emotional"[Title/Abstract] | 0 |
| #8 | | "depression emotional"[Title/Abstract] | 169 |
| #7 | | "Emotional Depression"[Title/Abstract] | 52 |
| #6 | | "symptoms depressive"[Title/Abstract] | 531 |
| #5 | | "symptom depressive"[Title/Abstract] | 5 |
| #4 | | "Depressive Symptom"[Title/Abstract] | 3,074 |
| #3 | | "Depressive Symptoms"[Title/Abstract] | 54,784 |
| #2 | | "Depressions"[Title/Abstract] | 5,850 |
| #1 | | "Depression"[MeSH Terms] | 132,415 |
| Source: Embase (Searched on: 28 September 2021) | | | |
| No. | Query | | Results |
| #19 | #14 AND #18 | | 5451 |
| #18 | #15 OR #16 OR #17 | | 377220 |
| #17 | 'multivessel coronary artery disease':ab,ti | | 2199 |
| #16 | 'coronary disease':ab,ti | | 22418 |
| #15 | 'coronary artery disease'/exp | | 369546 |
| #14 | #1 OR #2 OR #3 OR #4 OR #5 OR #6 OR #7 OR #8 OR #9 OR #10 OR #11 OR #12 OR #13 | | 549307 |
| #13 | 'parental depression':ab,ti | | 721 |
| #12 | 'mental depression':ab,ti | | 550 |
| #11 | 'depressive syndrome':ab,ti | | 1747 |
| #10 | 'depressive symptom':ab,ti | | 3875 |
| #9 | 'depressive state':ab,ti | | 1723 |
| #8 | 'depressive personality disorder':ab,ti | | 116 |
| #7 | 'depressive illness':ab,ti | | 4422 |
| #6 | 'depressive episode':ab,ti | | 8181 |
| #5 | 'depressive disorder':ab,ti | | 42987 |
| #4 | 'depressive disease':ab,ti | | 359 |
| #3 | 'clinical depression':ab,ti | | 3512 |
| #2 | 'central depression':ab,ti | | 651 |
| #1 | 'depression'/exp | | 545158 |
| Source: Cochrane Library (Searched on: 28 September 2021) | | | |
| ID | Search | | |
| #1 | MeSH descriptor: [Depression] explode all trees | | |
| #2 | ("Depression, Emotional"):ti,ab,kw (Word variations have been searched) | | |
| #3 | ("Depressive Symptoms"):ti,ab,kw (Word variations have been searched) | | |
| #4 | ("Symptoms, Depressive"):ti,ab,kw (Word variations have been searched) | | |
| #5 | ("Emotional Depressions"):ti,ab,kw (Word variations have been searched) | | |
| #6 | ("Depressions, Emotional"):ti,ab,kw (Word variations have been searched) | | |
| #7 | ("Symptom, Depressive"):ti,ab,kw (Word variations have been searched) | | |
| #8 | ("Depressive Symptom"):ti,ab,kw (Word variations have been searched) | | |
| #9 | ("Emotional Depression"):ti,ab,kw (Word variations have been searched) | | |
| #10 | ("Depressions"):ti,ab,kw (Word variations have been searched) | | |
| #11 | ("depression disorder"):ti,ab,kw (Word variations have been searched) | | |
| #12 | (" Neurosis, Depressive"):ti,ab,kw (Word variations have been searched) | | |
| #13 | ("Neuroses, Depressive"):ti,ab,kw (Word variations have been searched) | | |
| #14 | ("Depressive Neurosis"):ti,ab,kw (Word variations have been searched) | | |
| #15 | ("Depressive Disorders"):ti,ab,kw (Word variations have been searched) | | |
| #16 | ("Disorders, Depressive"):ti,ab,kw (Word variations have been searched) | | |
| #17 | ("Disorder, Depressive"):ti,ab,kw (Word variations have been searched) | | |
| #18 | ("Depressive Neuroses"):ti,ab,kw (Word variations have been searched) | | |
| #19 | ("Neurotic Depressions"):ti,ab,kw (Word variations have been searched) | | |
| #20 | ("Neurotic Depression"):ti,ab,kw (Word variations have been searched) | | |
| #21 | ("Depression, Neurotic"):ti,ab,kw (Word variations have been searched) | | |
| #22 | ("Depressions, Neurotic"):ti,ab,kw (Word variations have been searched) | | |
| #23 | ("Endogenous Depressions"):ti,ab,kw (Word variations have been searched) | | |
| #24 | ("Endogenous Depression"):ti,ab,kw (Word variations have been searched) | | |
| #25 | ("Depressions, Endogenous"):ti,ab,kw (Word variations have been searched) | | |
| #26 | ("Depression, Endogenous"):ti,ab,kw (Word variations have been searched) | | |
| #27 | ("Syndrome, Depressive"):ti,ab,kw (Word variations have been searched) | | |
| #28 | ("Depressive Syndromes"):ti,ab,kw (Word variations have been searched) | | |
| #29 | ("Depressive Syndrome"):ti,ab,kw (Word variations have been searched) | | |
| #30 | ("Syndromes, Depressive"):ti,ab,kw (Word variations have been searched) | | |
| #31 | ("Unipolar Depressions"):ti,ab,kw (Word variations have been searched) | | |
| #32 | ("Depressions, Unipolar"):ti,ab,kw (Word variations have been searched) | | |
| #33 | ("Unipolar Depression"):ti,ab,kw (Word variations have been searched) | | |
| #34 | ("Depression, Unipolar"):ti,ab,kw (Word variations have been searched) | | |
| #35 | ("Melancholias"):ti,ab,kw (Word variations have been searched) | | |
| #36 | ("Melancholia"):ti,ab,kw (Word variations have been searched) | | |
| #37 | #1 OR #2 OR #3 OR #4 OR #5 OR #6 OR #7 OR #8 OR #9 OR #10 OR #11 OR #12 OR #13 OR #14 OR #15 OR #16 OR #17 OR #18 OR #19 OR #20 OR #21 OR #21 OR #23 OR #24 OR #25 OR #26 OR #27 OR #28 OR #29 OR #30 OR #31 OR #32 OR #33 OR #34 OR #35 OR #36 | | |
| #38 | MeSH descriptor: [Coronary Disease] explode all trees | | |
| #39 | ("Coronary Diseases"):ti,ab,kw (Word variations have been searched) | | |
| #40 | ("Coronary Heart Disease"):ti,ab,kw (Word variations have been searched) | | |
| #41 | ("Diseases, Coronary"):ti,ab,kw (Word variations have been searched) | | |
| #42 | ("Disease, Coronary"):ti,ab,kw (Word variations have been searched) | | |
| #43 | ("Disease, Coronary Heart"):ti,ab,kw (Word variations have been searched) | | |
| #44 | ("Coronary Heart Diseases"):ti,ab,kw (Word variations have been searched) | | |
| #45 | ("Diseases, Coronary Heart"):ti,ab,kw (Word variations have been searched) | | |
| #46 | ("Heart Diseases, Coronary"):ti,ab,kw (Word variations have been searched) | | |
| #47 | ("Heart Disease, Coronary"):ti,ab,kw (Word variations have been searched) | | |
| #48 | #38 OR #39 OR #40 OR #41 OR #42 OR #43 OR #44 OR #45 OR #46 OR #47 | | |
| #49 | #37 AND #48 | | |
