# Supplementary material for: Depression increased risk of coronary heart disease: A meta-analysis of prospective cohort studies
Source: Front Cardiovasc Med. 2022 Aug 30;9:913888. doi: 10.3389/fcvm.2022.913888 (PMC9468274; doi:10.3389/fcvm.2022.913888)
Supplement: Supplementary Table 2 — Detailed scores of NOS for all eligible studies. [file Table_2.DOCX]

| Studies | Representativeness of the exposed cohort | Selection of non-exposed cohort | Ascertainment of exposure factor | Demonstration that outcome of interest was not present at start of study | Comparability of cohorts on the basis of the design or analysis (★★) | Evaluation of outcome | Was follow-up long enough for outcomes to occur | Adequacy of follow-up of cohorts | Quality Scores |
| --- | --- | --- | --- | --- | --- | --- | --- | --- | --- |
| Hawkins et al 2014 | ★ | ★ | ★ | ★ | ★★ | ★ | ★ | - | 8 |
| Brunner et al 2014 | × | ★ | ★ | - | ★★ | ★ | ★ | ★ | 7 |
| Rahman et al 2013 | ★ | ★ | ★ | ★ | ★★ | ★ | × | × | 7 |
| Huang et al 2013 | ★ | ★ | ★ | ★ | ★★ | × | ★ | ★ | 8 |
| Sun et al 2013 | ★ | ★ | ★ | ★ | ★★ | ★ | ★ | ★ | 9 |
| Pe´quignot et al 2013 | ★ | ★ | ★ | - | ★★ | ★ | ★ | - | 7 |
| Majed et al 2012 | × | ★ | ★ | ★ | ★★ | - | ★ | ★ | 7 |
| Brown et al 2011 | × | ★ | ★ | ★ | ★★ | ★ | ★ | - | 7 |
| Janszky et al 2010 | × | ★ | ★ | ★ | ★★ | × | ★ | - | 6 |
| Nabi et al 2010 | ★ | ★ | ★ | ★ | ★★ | ★ | ★ | ★ | 9 |
| Davidson et al 2009 | ★ | ★ | ★ | ★ | ★★ | ★ | × | - | 7 |
| Whang et al 2009 | × | ★ | ★ | ★ | ★★ | ★ | ★ | - | 7 |
| Ahto et al 2007 | ★ | ★ | ★ | - | ★★ | ★ | ★ | - | 7 |
| Kamphuis et al 2006 | ★ | ★ | ★ | - | ★★ | ★ | ★ | ★ | 8 |
| Wulsin, et al 2005 | ★ | ★ | ★ | ★ | ★★ | - | ★ | - | 7 |
| Gump et al 2005 | ★ | ★ | ★ | ★ | ★★ | ★ | ★ | ★ | 9 |
| Marzari et al 2005 | ★ | ★ | ★ | - | ★★ | ★ | × | - | 6 |
| Ferketich et al 2000 | ★ | ★ | ★ | ★ | ★★ | ★ | ★ | - | 8 |
| Sesso et al 1998 | × | ★ | ★ | ★ | ★★ | ★ | ★ | ★ | 8 |
| Jiang et al 2018 | ★ | ★ | ★ | ★ | ★ | × | ★ | - | 6 |
| Mendes et al 1998 | ★ | ★ | ★ | ★ | ★★ | ★ | ★ | - | 8 |
| Péquignot et al 2016 | ★ | ★ | ★ | ★ | ★★ | ★ | ★ | ★ | 9 |
| O’Neil et al 2016 | ★ | ★ | ★ | × | ★★ | × | ★ | × | 6 |
| Sims et al 2015 | ★ | ★ | ★ | ★ | ★★ | ★ | ★ | - | 8 |
| O’Brien et al 2015 | ★ | ★ | ★ | ★ | ★★ | ★ | ★ | - | 8 |
| Ford et al 1998 | × | ★ | ★ | - | ★★ | ★ | ★ | - | 6 |

Notes: “★” represents 1 point, “×” represents 0 point, and “—” represents uncertain points.

Abbreviation: NOS, Newcastle–Ottawa Scale.
